# Predictors of exclusive breastfeeding knowledge and intention to or practice of exclusive breastfeeding among antenatal and postnatal women receiving routine care: a cross-sectional study 

**DOI:** 10.1186/s13006-018-0154-0

**Published:** 2018-03-02

**Authors:** Thomas Senghore, Tobiloba Alex Omotosho, Omar Ceesay, Daisy Clara H. Williams

**Affiliations:** Department of Nursing and Reproductive Health, School of Medicine and Allied Health Sciences, University of The Gambia, P.O. Box 1646, Independence Drive, Banjul, The Gambia

**Keywords:** Exclusive breastfeeding, Knowledge, Intention, Practice, Predictors, The Gambia

## Abstract

**Background:**

Despite consistent evidence showing the importance of exclusive breastfeeding (EBF) for six months, it remains a sub-optimal practice in The Gambia. This study aimed at investigating the determinants of EBF knowledge and intention to or practice of EBF.

**Methods:**

A cross-sectional study was conducted among 334 women receiving care at the Edward Francis Small Teaching Hospital (EFSTH) from December 2015 to February 2016. Using a structured interviewer-administered questionnaire, knowledge on EBF was determined and scored. Participants scoring above or equal to the median were determined to have sufficient EBF knowledge. Multivariate logistic regression analyses were used to identify predictors of EBF knowledge and intention to or practice of exclusive breastfeeding.

**Results:**

The proportion of women with sufficient exclusive breastfeeding knowledge and intended to or practice EBF were 60.2% and 38.6% respectively, while only 34.4% received EBF counseling. Earning ≥1500 GMD monthly (Adjusted Odds Ratio [aOR] 1.98; 95% Confidence Interval [Cl] 1.24, 3.16), having positive attitude (aOR 2.40; 95% Cl 1.40, 4.10) and partner supporting EBF (aOR 2.18; 95% Cl 1.23, 3.87) predicted sufficient EBF knowledge. Mothers aged 26–34 years (aOR 0.50; 9 5% Cl 0.31, 0.82) and EBF counseling (aOR 2.68; 95% Cl 1.68, 4.29) significantly associated with intention to or practice of exclusive breastfeeding.

**Conclusion:**

In conclusion, improving EBF rates will, therefore, require improved access to information on EBF targeting low socio-economically disadvantaged and older mothers. In addition, emphasis on strengthening the ongoing EBF counseling already within the health system is required.

## Background

Exclusive breastfeeding (EBF), remains the optimal method of infant feeding [1] with major benefits for both mother and child [2–6]. It is estimated to improve the quality of lives of millions of children and prevent over 800, 000 under-five deaths annually if scaled up to near universal level [6–8]. Despite these substantial health benefits, the practice of EBF remains far sort of the level recommended by the World Health Organization (WHO), especially in developing countries where the burden associated with suboptimal infant feeding are high [9]. Globally, it is estimated that about 38% of infants 0 to 6 months of age are exclusively breastfed [10]. In Sub-Saharan Africa (SSA) where the practice of breastfeeding is common, EBF rates vary widely and range from 87.3% in Rwanda to 17% in Nigeria [11].

In 2003, WHO and UNICEF developed the Global Strategy for Infant and Young Child Feeding (IYCF) [12, 13]. In line with these strategies, the government of The Gambia through the National Nutritional Agency (NaNA) instituted policies to promote optimal infant feeding practices including strengthening and expanding the Baby Friendly Community and Hospital Initiative strategy, capacity building of healthcare providers on infant and young child feeding, enforcement of the Breastfeeding regulation 2006 and increasing awareness of legislators, policymakers and the public on the importance of optimal infant and young child feeding [14]. Despite the benefits and efforts to promote optimal infant feeding practices, EBF remains sub-optimally practiced in The Gambia. As highlighted in the 2013 Gambia Demographic and Health Survey (GDHS) report, only 46.8% of the infants were exclusively breastfed [15]. Obviously, this fall sort of the 90% coverage required in order to benefit from an 11.6% reduction of child deaths [8, 16]. On the other hand, infant and under-five mortality continue to be a major health problem in the Gambia. It has been estimated that 34 infants and 54 under-5 children per 1000 live births died in 2013 [15]. Hence understanding the factors that influence EBF is essential to help develop strategies for promoting the practice of EBF and by extension reduction in infant and child mortality.

A mother’s decision to practice EBF for the first six months is affected by several factors, including sociodemographic, maternal and child-related factors [17–20]. Breastfeeding knowledge including EBF knowledge also plays an important role in a mother’s ability to make an informed decision to EBF her infant. Evidence from developing countries has shown that mothers with better EBF knowledge are more likely to practice exclusive breastfeeding [21–23]. The knowledge about EBF is also influenced by several factors such as counseling on EBF, the number of children and intention to EBF [24, 25]. Several studies have investigated factors that influence EBF practice in SSA; however, findings vary between and within countries. For example, in Nigeria, maternal education, socioeconomic class, mode of delivery and infant’s first feed were associated with EBF in the South-east [20] while in the north-central region, prenatal EBF intention was the strongest predictor [26]. In Northwest Ethiopia, employment, income, health facility delivery and attendance of postnatal and antenatal clinics were identified as predictors of EBF [17]. A study in northwestern Tanzania concluded that advanced maternal age, knowledge of duration and advantages of EBF were found to predict EBF [21]. Similarly, in Ghana, higher education, baby younger than three months and high knowledge on EBF were determinants of EBF among rural lactating mothers [22]. Although documented evidence in The Gambia on predictors of EBF are scanty and old, qualitative studies suggest that strong cultural and traditional beliefs and inadequate information on the importance of EBF were attributed to the low rate of exclusive breastfeeding [27, 28].

Given the varied factors influencing the practice of EBF across different settings, setting-specific data are therefore necessary in order to develop tailor-made programs geared towards addressing the problem of suboptimal infant feeding. To promote EBF, the determinants of EBF must be identified and understood to develop appropriate policies to improve the situation of EBF. In The Gambia little is known about the factors that predict EBF knowledge and practice of EBF. This study aimed to investigate the predictors of EBF knowledge and intention to or practice of EBF among antenatal and postnatal mothers receiving routine care in a teaching hospital.

## Methods

### Study area and participants

This cross-sectional study was carried out at the Edward Francis Small Teaching Hospital (EFSTH) polyclinic in Banjul, The Gambia from 16th December 2015 to 8th February 2016. A wide range of services is offered by the clinic that includes but not limited to maternal and child health, family planning, immunization, health promotion and general out-patient care. For this study, only mothers attending antenatal and child health services with pregnancies over four weeks of gestation and postnatal mothers with children less than one year of age were included. Mothers with pregnancy complications or other comorbidities and children presenting specific feeding problems (cleft palate or lip and severely ill) were not legible to participate.

### Recruitment and data collection

Recruitment and data collection was done by student nurses trained on administering the questionnaire in major local languages. The clinic was visited on each clinic day and while mothers were waiting to receive care, they were approached to participate in the study. All participants arriving at the clinic between 08.30 am (clinic start) to 14.00 (clinic end) were contacted to participate in a successive manner. Those that consented to participate and were eligible were taken to a secluded area and after undergoing the consenting process, they were administered a face-to-face questionnaire in their local language if the participant does not understand the English language. All participants provided written informed consent.

### Measurements

The questionnaire which was adopted from previous instruments [22, 29, 30] was tested for reliability using test-retest while validity was established by breastfeeding experts. Following pilot testing in 20 antenatal and postnatal mothers, it was revised and then finalized for use. The instrument consisted of questions covering sociodemographic, reproductive health characteristics, knowledge on EBF, attitude towards EBF and intention to EBF (for antenatal mothers) or practice of EBF (for postnatal mothers). The Knowledge scale of the questionnaire consisted of 36 questions assessing mothers’ understanding and intellectual capacity to recall several aspects of EBF such as when to start breastfeeding after delivery, how often and for how long to breastfeed, benefits of the first milk (colostrum), how to improve breast milk supply, benefits of EBF for the first six months to the mother and child as well as the potential risks exposed to the child if not exclusively breastfed. Each correct response was accorded a point and no point in the case of a wrong response. Based on correctly answered questions, knowledge scores were obtained by summing up all correctly answered questions for each mother. A score of 29, which corresponds to the median, was used as the cut-off. Those scoring below the median were determined to have insufficient knowledge while scoring above or equal to the median was considered sufficient knowledge.

The attitude scale of the questionnaire consisted of five questions that sought to determine the mother’s feeling on negative cosmetic effects of EBF, whether it is an indication of poverty, ease, and convenience of EBF and confidence in expressing breast milk for the infant. Each attitude item had two responses (true or false), and depending on the question structure a response indicating a desire to EBF was considered positive attitude and scored one point. All points were summed to form an attitude score. Women scoring less than three points were considered to have less positive attitude, while those scoring three points and above were considered to have a positive attitude. They were also asked if they have ever had any counseling on EBF, who in their family supports EBF and their source of knowledge on exclusive breastfeeding. EBF was defined as per WHO [1] and the intention to EBF was defined as the planned length of EBF measured using the infant feeding intention scale [30].

### Sampling and sample size

A successive sampling design was used to sample participants. The required number of subjects ‘*n*’ for the study was estimated at 345 using the formula *n* = (*Z*^2^ × *P* × *Q*)/*D*^2^ where ‘Z’ is the critical value and for a two-tail test, is equal to 1.96, ‘*p*’ is the estimated proportion of mothers who EBF their infants for six months in the study setting population, which was considered to be slightly less than the national rate based on the latest GDHS [15] and was estimated at 34%. ‘Q’ is the proportion of mothers that do not EBF their infants (1 − *P*) which is 66% and ‘D’ is the accepted margin of error (0.05). A 2% contingency was added to account for any incomplete data making up a total of 352. An equal proportion of antenatal and postnatal women were recruited. About 5% of the sampled participants were dropped due to incomplete data and language barrier, leaving a final sample of 334 for final analysis.

### Data analysis

Data were analyzed using SPSS software for windows 20.0 (SPSS, Chicago, IL, USA). Baseline characteristics were summarized as mean and standard deviation for continuous variables and frequency and percentages for categorical variables. Unconditional binary logistic regression models were used to estimate the odds ratio (OR) and their 95% confidence intervals (Cls) to assess the univariate association between predictors and knowledge of EBF as well as the intention to or practice of EBF. Adjusted odds ratios (aOR) and 95% Cls were estimated in a multivariate logistic regression model that included only variables significant in the univariate model. A *p* <  0.05 was considered to be statistically significant.

## Results

### Sociodemographics, reproductive health characteristics, knowledge, attitude, and intention to or practice of EBF among mothers

A total of 334 women (166 antenatal and 168 postnatal) who met the inclusion criteria took part in the study. Table 1 shows the sociodemographics, reproductive health characteristics, knowledge, attitude, and intention to or practice of EBF of women. Most (160, 47.9%) of the mothers were below the age of 26 years, had secondary school education (153, 45.8%), were self-employed (232, 69.5%), and earned 1500 Gambian Dalasi (GMD) or more monthly. The majority of women had 3–5 children (117, 35.0%), were in their second trimester of pregnancy (76, 45.8%) and reported they had not received any counseling on EBF (219, 65.6%). The mean and median knowledge scores are 28.9 (Standard deviation: 3.7) and 29.3 respectively. About 63 % (201, 62.9%) of the women were considered to have sufficient knowledge of EBF; over three-quarters (269, 76.3%) had a good attitude towards EBF, 80.5% (*n* = 269) reported that their partners support EBF and 38.6% (*n* = 129) reported that they intend to or practiced exclusive breastfeeding.

**Table 1:** Sociodemographics, reproductive health characteristics, knowledge, attitude and intention to or EBF among women

Variable	*n* (%) (*n* = 334)
Age (*years)*	
≤ 25	160 (47.9)
26–34	150 (44.9)
≥ 35	24 (7.2)
Education	
Non-formal	87 (26.0)
Primary	71 (21.3)
Secondary	153 (45.8)
Tertiary	23 (6.9)
Occupation	
Self-employed	232 (69.5)
Formally employed	52 (15.6)
Unemployed	50 (15.0)
Monthly income *(GMD)*	
< 1500	143 (42.8)
≥ 1500	191 (57.2)
Pregnancy status	
Antenatal	166 (49.7)
Postnatal	168 (50.3)
Parity	
None	114 (34.1)
1–2	80 (24.0)
3–5	117 (35.0)
> 5	23 (6.9)
Gestational age *(n = 166)*	
1st Trimester	23 (13.8)
2nd Trimester	76 (45.8)
3rd Trimester	67 (40.4)
Counseling on EBF	
No	219 (65.6)
Yes	115 (34.4)
Attitude	
Less positive	79 (23.7)
Positive	255 (76.3)
Knowledge	
Insufficient	133 (39.8)
Sufficient	201 (60.2)
Partner supports EBF	
No	65 (19.5)
Yes	269 (80.5)
Intention to or EBF	
No	205 (61.4)
Yes	129 (38.6)

*EBF* Exclusive breastfeeding, *GMD* Gambian Dalasi

### Predictors of sufficient knowledge on EBF

In the univariate analysis earning 1500 GMD or more monthly (OR 2.05; 95% Cl 1.31, 3.21; *p* = 0.002), counseling on EBF (OR 1.85; 95% Cl 1.15, 2.98; *p* = 0.012), having a positive attitude towards EBF (OR 2.36; 95% Cl 1.40, 3.92; *p* = 0.001), partner supporting EBF (OR 2.20; 95% Cl 1.27, 3.81; *p* = 0.005) and intending to or practicing EBF (OR 1.65; 95% Cl 1.04, 2.62; *p* = 0.032) were significantly associated with an increased odds of having sufficient knowledge on EBF. In comparison, to earning < 1500 GMD, no counseling on EBF, less positive attitude, partner does not support EBF and those who do not intend to or practice EBF respectively. In the multivariate analysis, after adjusting for potential confounders, earning 1500 GMD or more monthly (aOR 1.98; 95% Cl 1.24, 3.16; *p* = 0.004), having a positive attitude towards EBF (aOR 2.40; 95% Cl 1.40, 4.10; *p* = 0.003) and partner supporting EBF (aOR 2.18; 95% Cl 1.23, 3.87; *p* = 0.008) remain significantly associated with increased odds of having sufficient knowledge on exclusive breastfeeding (Table 2).

**Table 2:** Factors associated with sufficient EBF knowledge among women

	Unadjusted		Adjusted	
Variable	OR (95% Cl)	*p*-value	^a^OR (95% Cl)	*p*-value^b^
Monthly income *(GMD)*				
< 1500	1		1	
≥ 1500	2.05 (1.31, 3.21)	0.002	1.98 (1.24, 3.16)	0.004
Counseling on EBF				
No	1		1	
Yes	1.85 (1.15, 2.98)	0.012	1.53 (0.92, 2.56)	0.104
Attitude				
Less positive	1		1	
Positive	2.36 (1.40, 3.92)	0.001	2.40 (1.40, 4.10)	0.003
Partner supports EBF				
No	1		1	
Yes	2.20 (1.27, 3.81)	0.005	2.18 (1.23, 3.87)	0.008
Intention to or EBF				
No	1		1	
Yes	1.65 (1.04, 2.62)	0.032	1.43 (0.87, 2.36)	0.154

*OR* Odds ratio, *Cl* confidence interval, *EBF* Exclusive breastfeeding, *GMD* Gambian Dalasi

^a^Adjusted for family income, counseling on EBF, intention to or practice EBF, Attitude and Husband supports EBF

^b^Significant at *p* <  0.05

### Predictors of intention to or practice of EBF

Factors associated with intention to or practice of EBF in the univariate analysis included age, counseling on EBF and knowledge on EBF. Women aged 26–34 years (OR 0.57; 95% Cl 0.36, 0.91; *p* = 0.018) were at decreased odds of intending to or practicing EBF compared to women ≤25 years of age. Those who receive counseling on EBF (OR 2.81; 95% Cl 1.76, 4.48; *p* <  0.001) and had sufficient knowledge on EBF (OR 1.65; 95% Cl 1.04, 2.62; *p* = 0.032) were found to possess increased odds of intending to or practicing EBF compared to those with no counseling and insufficient knowledge on EBF. After adjusting for confounders mother’s age 26–34 years (aOR 0.50; 95% Cl 0.31, 0.82, *p* = 0.006) and counseling on EBF (aOR 2.68; 95% Cl 1.68, 4.29; *p* <  0.001) remain significantly associated with intention to or practice of exclusive breastfeeding. However, having sufficient knowledge of EBF (aOR 1.58; Cl 0.97, 2.56; *p* = 0.064) showed a borderline significant association with intention to or practice of exclusively breastfeed (Table 3).

**Table 3:** Factors associated with intention to or EBF among women

	Unadjusted		Adjusted__	
Variable	OR (95% Cl)	*p*-value	^a^OR (95% Cl)	*p*-value^b^
Age *(years)*				
≤ 25	1		1	
26–34	0.57 (0.36, 0.91)	0.018	0.50 (0.31, 0.82)	0.006
≥ 35	1.52 (0.64, 3.60)	0.341	1.14 (0.46, 2.82)	0.779
Education				
Non-formal	1			
Primary	0.55 (0.29, 1.16)	0.126		
Secondary	1.41 (0.82, 2.42)	0.210		
Tertiary	1.32 (0.52, 3.36)	0.557		
Monthly income (*GMD)*				
≤ 1500	1			
≥ 1500	1.46 (0.93, 2.29)	0.101		
Counseling on EBF				
No	1		1	
Yes	2.81 (1.76, 4.48)	< 0.001	2.68 (1.68, 4.29)	< 0.001
Knowledge of EBF				
Insufficient	1		1	
Sufficient	1.65 (1.04, 2.62)	0.032	1.58 (0.97, 2.56)	0.064

*OR* Odds ratio, *Cl* confidence interval, *EBF* Exclusive breastfeeding, *GMD* Gambian Dalasi

^a^Adjusted for age, counseling on EBF and Knowledge on EBF

^b^Significant at *p* < 0.05

## Discussion

Considering that EBF for the first six months of life remains suboptimally practiced in the Gambia, identifying predictors may help develop appropriate policy and interventions to increase the practice of exclusive breastfeeding. In this study, we investigated the predictors of knowledge and intention to or practice of exclusive breastfeeding. Our finding suggests that factors such as monthly income, attitude towards EBF and partner supporting EBF were significantly associated with sufficient knowledge on exclusive breastfeeding. While the intention to or practice of EBF was associated with age and counseling on exclusive breastfeeding.

We found a low rate of women who underwent breastfeeding counseling (34.4%) during the antenatal period. This finding is notable, given that counseling on EBF is recommended for all pregnant women as early as the first trimester. In fact when only antenatal mothers were considered, about 16% of the mothers who had two and more antenatal visits (74.7%) received counseling on EBF, many of them in their third trimester and beyond. This finding is similar to those in Tanzania [21, 24] and Ethiopia [31], where most women received antenatal care (96.7% and 78% respectively) but only a few (49% and 48% respectively) received counseling on infant feeding practices. The low rate of EBF counseling among these mothers implies that health staff are not taking advantage of the opportunity to educate mothers on the importance of EBF thereby making it difficult to improve uptake and adherence on exclusive breastfeeding. Further studies may be required to investigate the possible reasons for the low intake of EBF counseling. Thus, aiding efforts aimed at increasing the uptake of counseling on EBF. More than half (60.2%) of the participants were considered to have sufficient knowledge of EBF. This is remarkable given the low rate of mothers who received counseling on exclusive breastfeeding. Other sources of knowledge may have accounted for this proportion and in fact, the majority of the mothers (47%) reported family members as their major source of knowledge on exclusive breastfeeding. This implies that other sources of communication such as family members can be good sources of knowledge on exclusive breastfeeding. Evidence has shown that grandmother’s knowledge of infant and young child feeding practice (IYCF) correlates with a mother’s correct knowledge on IYCF [32]. Therefore, health education programs on EBF must be designed to reach a wider audience possibly through radio, television and the use of social media. The 60.2% proportion of sufficient knowledge in our study is consistent with findings from Moshi, Tanzania [24], but lower than findings from Nigeria [33], where 61.2% and 71.3% of mothers respectively had appropriate knowledge on optimal breastfeeding practice.

### Predictors of sufficient knowledge on EBF

In the multivariate logistic regression analysis, mother’s monthly income, attitude towards EBF and partner supporting EBF were found to be associated with sufficient EBF knowledge. Women who earned 1500 GMD monthly (which was approximately 40 USD) and more were about two times more likely to have sufficient knowledge on exclusive breastfeeding. Higher income earning women usually belong to a modernized society with greater access to information from the electronic and print media. Therefore, improved access to information is required especially targeting women of low socioeconomic class. Mothers with a positive attitude towards EBF were also found to have a higher odds of sufficient knowledge on exclusive breastfeeding. This may be because women with a more positive attitude on EBF are more open and receptive to information on exclusive breastfeeding. On the other hand, their knowledge may have lead to a more positive attitude towards exclusive breastfeeding. However, due to the cross-sectional nature of our data, we cannot establish any temporality. Women whose partners support EBF were also found to have over two times increased odds of having sufficient knowledge on exclusive breastfeeding. The possible reason for this may be that these partners were knowledgeable or had a positive attitude towards EBF and therefore more supportive in providing information to their spouses. Father’s improved knowledge and attitude towards infant feeding were not only found to correlate with mother’s breastfeeding knowledge but also the practice of exclusive breastfeeding [34, 35].

In our study, counseling on EBF and intention to or practice EBF were not associated with sufficient EBF knowledge. Contrary to our findings, Hashim et al., found counseling and intention to practice EBF to be significantly associated with appropriate breastfeeding knowledge among pregnant women in Tanzania [24]. Our findings of no statistically significant association between counseling and sufficient knowledge on EBF might be that information provided during counseling was suboptimal. As found in the Tanzania study, among women who received counseling on infant feeding the information given was suboptimal [24]. Another possibility could be that mothers may have just forgotten the information given to them during counseling, particularly for those who received counseling long time ago. Further studies may be required to evaluate content and methods used in counseling mothers.

### Predictors of intention to or practice of EBF

The study findings show that the proportion of women who intend to or practice EBF (38.6%) was low. Though similar to the 38% estimated globally [10], it is lower than the 47% national average for The Gambia [15]. However, this figure is higher than the 33.5% in Nnewi, south-eastern Nigeria [20], 24.1% in Tanga, north-eastern Tanzania [21] and 30% in Burkina Faso [36]. Our study was conducted within the capital city where the majority of the mothers were self-employed, which may have accounted for the low proportion of exclusive breastfeeding. Evidence shows that employed mothers are less likely to EBF their infants for six months [37, 38]. Dun-Dery et al. also reported a very low proportion (10.3%) of EBF among city-dwelling professional working mothers in Ghana [19]. The low proportion of EBF found in our study could also be attributed to the cultural beliefs and misconceptions held about EBF as reported previously in rural Gambian settings [27]. These results also reflect the wide variation within the countries. It is also possible that these differences might be due to the different methodologies used in estimating EBF rates in different studies [39]. Notwithstanding, the proportion of EBF found in both our study and that reported in the GDHS 2013 are far less than the 90% universal coverage necessary to substantially reduce under-five mortality [8]. Therefore, there is need to implement and intensify intervention programs that will promote EBF, even within urban settlements.

In the present study, the age of the mother was significantly associated with intention to or practice of exclusive breastfeeding. Mothers aged between 26 and 34 years were found to have about twice decreased likelihood to intend to or practice EBF compared to those age ≤ 25 years. In agreement with our study, previous evidence from developing countries shows that older women were less likely to practice exclusive breastfeeding [26, 37, 40]. This could be explained by the fact that mothers within this age bracket have better job opportunities and lack the time to EBF their infants. Although in The Gambia legislation exists offer nursing mothers six months paid maternal leave, self–employed mothers may not give themselves this six months employment break. As shown in our study majority of the women were self-employed. Therefore, specific programs need to be adopted for these women so as to enable them EBF their infants.

Several interventions have constantly proven that education and support from a healthcare provider improve EBF rates [41, 42]. In our study, we found mothers who received counseling on EBF to be over twice more likely to intend to or practice EBF compared to those who did not receive counseling, that is consistent with many other studies [19, 25, 31, 43]. Counseling provides an opportunity for a face-to-face interaction with the mother, to receive tailor-made information in response to her own needs and therefore has the potential to influence an individual’s decision-making process, especially if provided by a knowledgeable and supportive counselor. Unfortunately, our data show that a very small proportion of women received any counseling on breastfeeding. A possible reason for this might be that healthcare staff might be overwhelmed by the high workload and might possibly overlook the need for counseling. It may also be that healthcare providers may think these women already have enough knowledge on exclusive breastfeeding. An appropriate strategy needs to be put in place to ensure that all antenatal mothers receive counseling. A possible way could be the provision of specifically trained breastfeeding counselors whose prime responsibility will be to counsel mothers. Other ways could be home-based and with peer counseling.

Another factor that was found to associate with the intention to or practice of EBF was knowledge on exclusive breastfeeding. Mothers with sufficient knowledge on EBF were more likely than their counterparts with insufficient knowledge on EBF to intend to or practice exclusive breastfeeding. However, this result attained borderline statistical significance and must be interpreted with caution. Further studies may be required to confirm these results. However, many other studies reported high maternal knowledge to associate with high EBF rates [22, 43, 44], and the reverse for low maternal EBF knowledge [23, 45].

This study is not without limitations. Its cross-sectional nature makes it difficult to infer causality. We cannot also exclude the possibility of recall bias as postnatal women may not recall when they introduced liquids or other solids. However, the study was restricted to only women with children age less than one year, therefore, limiting any form of recall bias for determining if they practice EBF for six months. Another issue is the self-report information which may be subject to social desirability bias. For the antenatal women, the intention to exclusively breastfeed was used as a proxy to determine EBF practice. These women may not necessarily follow what they say they intend to do, which may over or underestimate EBF rate in our study. However, a very strong desire to EBF has been found to correlate with EBF up to six months [46]. In addition, the items on the questionnaire have been validated thus improving the credibility of our findings [30]. Finally, this study only involved women receiving routine care at a tertiary teaching hospital in the capital city and therefore cannot be generalized to the entire population of antenatal and postnatal Gambian women. Despite these limitations, our findings provide insight into the predictors of EBF in The Gambia. This information could be useful for policy and interventions aimed at improving EBF rates.

## Conclusion

The results of this study indicate that 60.2% of the mothers had sufficient knowledge on EBF with only 34.4% receiving counseling on EBF and 38.6% intended to or practice exclusive breastfeeding. Mothers’ income, attitude towards EBF and partner support for EBF were predictors of sufficient EBF knowledge while mother’s age and counseling on EBF predicted intention to or practice of exclusive breastfeeding. Attempts at improving women’s knowledge on EBF should target socioeconomically disadvantaged mothers and family members, especially partners. Interventions to improve EBF rates must focus on older mothers with emphasis on improvement and strengthening of the ongoing EBF counseling within the health system.

## Abbreviations

aOR: Adjusted odds ratio
Cl: Confidence interval
EBF: Exclusive breastfeeding
GDHS: Gambian Demographic and Health Survey
GMD: Gambian dalasi
IYCF: Infant and Young Child’s Feeding
NaNA: National Nutrition Agency
OR: Odds ratio
SSA: Sub-Saharan Africa
UNICEF: United Nations Children’s Fund
USD: United States dollar
WHO: World Health Organization
